# Gurus and Griots: Revisiting the research informed consent process in rural African contexts

**DOI:** 10.1186/s12910-021-00659-7

**Published:** 2021-07-23

**Authors:** Richard Appiah

**Affiliations:** College of Health Sciences, University of Ghana, Korle-Bu, P. O. Box KB 143, Accra, Ghana

**Keywords:** Research informed consent process, Community-based participatory action research, Ethical and cultural considerations, Rural Africa, Ghana

## Abstract

**Background:**

Researchers conducting community-based participatory action research (CBPAR) in highly collectivistic and socioeconomically disadvantaged community settings in sub-Saharan Africa are confronted with the distinctive challenge of balancing universal ethical standards with local standards, where traditional customs or beliefs may conflict with regulatory requirements and ethical guidelines underlying the informed consent (IC) process. The unique ethnic, socioeconomic, and cultural diversities in these settings have important implications for the IC process, such as individual decisional autonomy, beneficence, confidentiality, and signing the IC document.

**Main text:**

Drawing on insights and field observations from conducting CBPARs across several rural, highly communal, low literate, and low-income communities in Ghana, we discuss some theoretical, ethico-cultural, and methodological challenges associated with applying the universal, Western individualistic cultural value-laden IC process in sub-Saharan Africa. By citing field situations, we discuss how local cultural customs and the socioeconomic adversities prevalent in these settings can influence (and disrupt) the information disclosure process, individual decisional authority for consent, and voluntariness. We review the theoretical assumptions of the Declaration of Helsinki’s statement on IC and discuss its limitations as an ultimate guide for the conduct of social science research in the highly communal African context. We argue that the IC process in these settings should include strategies directed at preventing deception and coercion, in addition to ensuring respect for individual autonomy. We urge Universities, research institutions, and institutional review boards in Africa to design and promote the use of context-appropriate ethical IC guidelines that take into consideration both the local customs and traditional practices of the people as well as the scientific principles underpinning the universal IC standards.

**Conclusion:**

We recommend that, rather than adopt a universal one-size-fits-all IC approach, researchers working in the rural, highly collectivistic, low literate, socioeconomically disadvantaged settings of sub-Saharan Africa should deeply consider the roles and influence of cultural values and traditional practices on the IC and the research process. We encourage researchers to collaborate with target communities and stakeholders in the design and implementation of context-appropriate IC to prevent ethics dumping and safeguard the integrity of the research process.

## Background

When conducting research with populations from highly collectivistic, low literate, and socioeconomically disadvantaged settings in developing countries, researchers, often unintentionally, apply the universal ethical guidelines (UEGs; e.g., the informed consent [IC]) to the local context, without much consideration of the distinct cultural and socioeconomic circumstances of the people. Although the UEGs work reasonably well in all contexts, researchers often do not recognise the extent to which the IC rests on the cultural values and traditional practices of the people. In the rural, more communal context of Ghana, and sub-Saharan Africa more generally, cultural norms have significant influence on the social and behavioural expressions of the people. In these settings, the process to obtain IC, ensure protection of privacy and confidentiality, and establish trusting relationships between researchers and participants takes on greater complexity [1]. One of the reasons for this, we contend, is that, largely, the UEGs adopt an individual-based consent model, where norms of decision-making emphasise respect for individual autonomy [2, 3]. However, in most close-knitted, low literate, socioeconomically disadvantaged, and highly collectivistic settings in sub-Saharan Africa, residents are considered as members of the society rather than as autonomous individuals [1, 4], and research activities ought to be firstly approved by gatekeepers of the communities and households (i.e., chiefs, elders, heads of households, husbands) before members can be recruited [1, 5]. Theoretically, these cultural norms and values undermine the principle of individual autonomy.

To explore this, first, we begin with a brief review of the literature on the role of culture and cultural norms in the IC process, from a global perspective. We then provide a brief review of the challenges with using the universal IC framework in the more collectivistic African setting. Next, we draw on insights from our previous community-based participatory action research (CBPAR) engagements to discuss the role of gatekeepers (i.e., chiefs, community elders, heads of households, and husbands of prospective female participants) in the IC process and how their roles conflict with the principles underpinning the universal IC process. Moving next to more socioeconomic factors, we identify and discuss how low literacy and poverty can also cause a conflict with the IC process in these settings. In both illustrations, we offer suggestions to prospective researchers to envision and manage these conflicts in a more appropriate way. Subsequently, we discuss the Declaration of Helsinki’s statement on the IC, its limitations and implications for social science research, and argue for a broader conceptualisation of the IC process to allow researchers to manage ethico-cultural conflicts with the IC more appropriately—as required or applicable in their study settings. In closing out, we urged Universities, research institutions, institutional review boards (IRBs), and other stakeholders to consider developing context-appropriate, diversified IC framework that takes into account the unique cultural and traditional practices of the target group as well as the principles underpinning the UEGs.

## Main texts

### Cultural factors and the IC process

Researchers and practitioners working with human subjects are required to adhere to specific international ethical research guidelines, such as the Belmont Report [6], the Nuremberg code [7], and the Declaration of Helsinki [8], which outline ethical principles to guide the conduct of medico-social research, including the processes involved in obtaining informed consent (IC) for participation in research. As the predominant ethical guideline for conducting research with human subjects, the Declaration of Helsinki ethical principles [8] include a discussion on the core values that underpin the IC process with an explicit proclamation for researchers to obtain IC from prospective participants (or their legally acceptable representatives) before they are recruited into a study. The IC provides opportunity for researchers to inform prospective participants about the objectives of the study, what is expected of study participants, the possible benefits and risks for partaking in the study, the processes instituted to ensure confidentiality and anonymity, voluntariness of participation, and how the findings would be communicated to participants and the general public [3]. The IC process upholds the individual’s decisional authority, and provides the necessary information to help the prospective participant to decide whether or not to participate in a research study or procedure [3, 5, 6]. The IC process is a continuous interactive engagement between the research team and the participants to resolve all concerns and ambiguities, and build a collaborative, trusting relationship [3, 5].

The question of whether the UEGs is applicable to all research contexts is the cause of considerable debate. A growing number of researchers working in highly communal, low literate, rural African settings report that the research process is significantly dictated by the cultural values and traditional practices prevailing in specific local settings, and are distinct from those in highly individualistic social settings. An impressive body of evidence exists to support this assertion in sub-Saharan Africa [9–12] and in studies conducted in Western countries with minority groups [13–15]. Although cultural values and norms are visibly ubiquitous and explicitly expressed in more collectivistic social settings (e.g., Africa and Asia), cultural factors have also been found to strongly influence participants’ understanding and awareness of the scientific research process [13], and had determined the language and communications tools appropriate for use in research for specific groups in more individualistic social settings [14, 16]. A growing number of global and multicultural body of work provide insights into the fundamental role and influence of cultural beliefs and practices on the IC process, particularly amongst participants from minority ethnic groups—who are also often from more collectivistically oriented societies. In the United States, cultural factors were implicated in how a sample of African Americans viewed their rights as research participants [17]. This was also the case among a sample of Asian American participants [18], as well as among a group of rural and peri-urban adult participants in Mali [19].

As clearly demonstrated by Chen et al. [20] in their study examining the challenges and solutions to conducting mental health research among Asian Americans, cultural factors can significantly influence participants’ preference for oral consent, instead of providing written informed consent. In some instances, researchers conducting multi-country and cross-cultural studies first needed to explore and acquaint themselves with existing cultural beliefs and ethico-cultural practices before commencing their research activities [21, 22]. Owing to cultural practices and low literacy, study participants from Navajo, a Native American people of the Southwestern United States, opted to thumbprint rather than sign on the consent form [23]. In this context, researchers observed that the IC process led to confusion, misperception, and embarrassment for study participants. In more communal African settings, the process for administering the IC could require a discussion with the entire family of the prospective participant, before the individual can sign the IC form [1, 24]. Study participants could also be coerced by the way and manner the research questions are framed [25], or when a community elder who wields some authority in the community takes on the role of an independent mediator [1].

Although the involvement of family members in the IC process tend to undermine the principle of autonomy or individual decisional authority, Marshall et al. [26] contends that their involvement in the decision-making process does not erode the probability that selected individuals will voluntarily take part in the research activity. Researchers hold the view that in highly communal social settings, it should be possible for other members to share their views on the IC process (i.e., communal decisions), and have a particular individual who agrees to participate to sign the form [1, 24]. Other cultural factors, such as the researchers’ level of knowledge about the cultural values and practices of the target population, and their capacity to identify and manage the ethico-cultural issues in specific research settings, have been noted [27, 28]. There are also manifold beliefs that hinder researchers’ efforts to obtain IC in highly religio-cultural settings. For instance, in Gabon [29] and the Gambia [30], the cultural belief that blood is a living, spiritual part of the individual, and that samples collected by researchers could be sold or used for charms—which can be employed to harm the donor—breed distrust toward clinical research.

### Challenges with the UEGs in the collectivistic African context

Although there is a growing effort within the global context to develop standardised guidelines to help researchers to envision and resolve possible ethical conflicts and to navigate the research process more effectively [31–34], what is lacking in these works is context-specific guidelines that propose specific solutions for researchers conducting CBPARs in highly collectivistic, low literate, and socioeconomically disadvantaged settings in sub-Saharan Africa. The majority of existing guidelines discuss ethical issues such as confidentiality, IC, deception, privacy, and anonymity from a general, global perspective, without recourse to possible sociocultural differences in study settings. There is presently a heightened interest to re-examine the use of the universal, individual-focused ethical guidelines, which is premised on Western individualistic social orientation, in non-Western, more collectivistic social contexts, and to adapt aspects of the UEGs (e.g., the IC) to the unique cultural and socioeconomic circumstances of participants [5, 9–12]. Largely, the universal IC guidelines adopt the individual-based consent model, where the decisional authority rests entirely on the individual [2, 3]. However, in most rural, highly collectivistic communities in sub-Saharan Africa, participating in a research—which ought to be firstly approved by the gatekeepers of the communities (i.e., chief and elders of the community)—is more or less an altruistic endeavour for the benefit of the community and society, rather than an individual’s private engagement. This elevated notion of ‘duty to others’ in this highly communitarian, culturally-adherent context, is comparable to the notion of ‘individual rights’ in Western ethics, where the emphasis is placed on individual rights, identity, and needs, over the community’s. Specifically, in settings where cultural values and practices emphasise oral rather than written agreements, and where the tribal chiefs and community leaders play central roles in the social life and decision-making process of the people, the use of (written) universal ethical documents, where the decisional authority rests on the individual (i.e., private sphere) rather than the community (i.e., public sphere), may be problematic.

Furthermore, in settings characterised by high levels of poverty and low literacy, it is possible that researchers and participants may share different views and beliefs about the IC process, due to the possible variations in their conceptualisation, comprehension, or interpretation of the problem under study or the research process [10]. Other inter-related issues, such as linguistic barriers to effective communication—especially when the consent forms are not translated into the native language of participants—can adversely affect participants’ understanding of the IC process. While it is desirable to have a universally-accepted guideline for obtaining IC, the diverse sociocultural perspectives between individualistic and collectivistic societies, and on beliefs about truth telling, disclosure of health-related information, and decisional authority with respect to the IC process, have warranted an open discussion on the need for research ethical guidelines to be adapted to the unique cultural and socioeconomic circumstances of participants [5, 9, 10]. Of note, the decision-making process by an individual in the rural, highly collectivistic social setting in Ghana and sub-Saharan Africa more generally, to participate in a research activity may often involve a discussion with family members, and possibly with some members of the community, before finalising their decision to participate or not [1].

The main argument supporting the universal IC is that it should be a necessary standard or criterion for prospective participants to exercise autonomy—freedom from controlling interference by others, as well as from their personal limitations—in their decision-making process about whether or not to agree to participate in a research activity [2, 35]. While this principle ensures that the decision lies entirely with the prospective participant, we find that it somewhat contradicts some other UEGs and may conflict with the local standards in some specific contexts. First, when applying the CBPAR approach, researchers often provide prospective participants an ample time (e.g., few days) to finalise their decisions about whether or not to participate in the study [36, 37]. Often, in social science research, the prospective participant may be asked to confer with a dedicated independent mediator or lead researchers to clarify their concerns. We note that in most rural, highly collectivistic social settings, individuals may also discuss aspects of their concerns with their family members, or even other members of the community, as part of the decision-making process. We find that while this process appears to contradict the basic principles of autonomy, it does not, necessarily, undermine the individual’s decisional power or the voluntariness of their participation. We compare and liken this illustration with the process used to  obtain IC in biomedical research, where guardians or legally acceptable representatives consent for vulnerable or incompetent prospective participants [38]. We however admit that when a prospective participant discusses the IC process with others, the nature of the discussion (i.e., in terms of the nature of the views and suggestions shared by the discussants) can possibly weigh in on the final decision of the prospective participant. But people—whether or not they are literate or from highly individualistic or collectivistic social settings—are rational beings and researchers must trust their final decisions. The commitment to solicit for views from family members or from other members of the community only goes to demonstrate and support the fundamental notion that people are social beings, especially those from highly collectivistic societies [4].

### Gatekeepers and the IC process in the collectivistic Ghanaian context

Working as a research implementation associate at Innovations for Poverty Action from March 2015 to November 2017 on the Escaping Poverty (EP) project [39], the author was privileged to lead the mental health (cognitive-behavioural intervention) arm of the EP project. The mental health arm sought to promote participants’ mental health, build their resilience, and increase their vocational productivity by helping them to develop skills in time management, goal setting, problem solving, and relationship and communication, and to enhance their cognitive abilities to help them to identify and challenge unhelpful thinking patterns. The author led the development of the intervention programme, recruited and trained session facilitators (lay counsellors), and supervised the community entry process, recruitment of participants, and implementation of the programme. The 12-session, two-hourly, once-weekly group-based intervention programme was administered to 1,572 rural adults selected from 165 communities across four regions of Ghana. This was also the case in another study—the author’s doctoral project—where he led the development of a 10-session multicomponent positive psychology intervention programme (the *Inspired Life Programme*; ILP [40]), recruited and trained session facilitators, and supervised the community entry and implementation processes in four rural communities in the Brong Ahafo region.

Each of these communities are governed by elected chiefs, his council of elders, and other community leaders. These leaders, who are often from a royal lineage, have the utmost responsibility of ensuring that community members uphold the cultural customs and traditional practices of the ethnic group. In these settings, the chiefs and community leaders serve as the gatekeepers to the community—who must be consulted for permission to conduct a research study. The community entry process in these communities follows similar pattern. Upon entry into the community, the research team first contacts a community leader, who welcomes the team and inform them about the customary rites required to visit the chief and his elders. For communities in northern Ghana, a calabash of *colanuts* or an amount of about GHS 40.00 (about $ 6.5) in a sealed envelope is presented. Some three or four bottles of schnapps (or its monetary equivalent) are required for communities in southern Ghana. The community elder leads the team to visit the chief and his elders to inform them about the research. The chief and elders may seek for further clarifications, such as why their community was selected for the study. A detailed discussion of the community entry process in the rural, low socioeconomic, highly collectivistic context of Ghana is presented elsewhere [1].

The cultural-laden community entry process in these settings has a few implications for the IC process. First, in most remote settings, the chief and elders, upon granting permission to the research team, have the duty to ask the town crier^1^ to inform community members about the presence of the researchers in the community and to ask members to participate in the study and to offer the researchers any assistance they may require with their research activities. This traditional obligation to inform members of the community about the research activity has the potential to coerce community members to participate in the study, considering that information about the study and request of support was authorised by the chief or community leaders. This form of coercion, albeit unintentionally, contradicts the principle of individual decisional authority. When the offer to inform members of the community is made, the researcher should express appreciation and respectfully explain to the chief and elders that such an arrangement can have adverse implications on the research by compelling members to participate, against their volition. It is important for the researcher to clarify to the chief and elders that there would be no need to pre-inform community members about the research by providing the same justification, even when the chief or elders do not discuss this intention at the meeting. Second, to ensure that the researchers have adequate understanding of the cultural customs and language of community members, an individual from the community may be trained to act as an *independent mediator* to usher the research team into the homes of community members and lead the IC process. This person also serves as a liaison, to whom selected members can later contact for clarity on aspects of the IC and research activities. Support may be solicited from the chief and elders to identify a suitable individual—who must know the community terrain, but must not wield authority in the community to influence members’ decision to participate. Although the independent mediator is an important part of the research team when conducting CBPAR [36, 37], it is difficult to ignore their influence on the IC process, given that prospective participants may rely on the response and information they provide to help them to finalise their decision of whether or not to participate in the study. 

One of the most significant stakeholders in the IC process in the rural, highly collectivistic social settings in sub-Saharan Africa is the head of the households. It is a cultural requirement, in many rural, highly collectivistic social settings in Ghana, to seek for approval from the head of a household before proceeding to invite a member of the household to participate in a research study. The head of the household—who is often the oldest male of the household—welcomes the team and asks for further clarifications about the study before granting approval. In the event that a married female needs to be recruited, there is need to obtain permission from the husband [1, 9, 10]. Generally, the research process, particularly the IC process, takes on greater complexity in the rural, highly communal context of Ghana, and sub-Saharan Africa more generally [1]. For instance, amongst the Kassena, Nankana, and Talensi ethnic groups of the Upper East region of Ghana, cultural norms forbid the head of the household to speak directly with the researcher in their first encounter. The research team first approaches a younger member of the household to discuss about the study and express their wish to recruit a member of the household. This information is passed on to the head of the household, who explains that he needs to ‘sleep over the matter’ and consult the ancestors and adult members of the household. Subsequently, a meeting is scheduled with the research team. After a series of questioning, the head of the household may approve the team’s request and may nominate members of the household to participate. Of note, the involvement of the chiefs and elders, heads of households, and husbands of female prospective participants as gatekeepers, as well as the role of the independent mediator as lead for the IC process, are strongly established cultural customs and traditional practices that cannot be circumvented. It is commonplace for individuals who do not come from large households, or who live alone, to first confirm with the researcher whether the chief and his elders have been consulted and given their approval to the study, before they consent to participate. In whichever form it takes, these customary practices undermine the pillars of autonomous choice, which is fundamental to the universal ethical practice for IC. By expressing their appreciation, the independent mediator and the research team carefully explain that the research process requires that each qualified member in the household be given the chance to be enrolled, in order to ensure fairness. Although the team may be granted the opportunity to randomly select members into the study, selected members may be more inclined to consent to participate, on the basis that approval has been granted by the head of the household and adult members.

Joining the wealth of literature and observations from our field experiences on the many ways that the use of the standard, universal ethical protocols for the IC process can go awry in rural, highly collectivistic, low literate African contexts, we recommend that researchers also conceptualise and implement strategies aimed at preventing deception and coercion of prospective participants. The roles of the chiefs and elders, heads of households, and husbands of female prospective participants as gatekeepers, as well as the independent mediator as lead for the IC process, are inherent cultural mechanisms to protect members from exploitation and deception. It is unethical, therefore, for researchers to attempt to circumvent the cultural customs or influence community members to change their values and practices in any way. An imposition of the researchers’ values and principles on local participants equates to *ethics dumping*, and has dire implications for the integrity of the research process—and the researchers’ own integrity. Researchers, before submitting their proposals to the IRBs to conduct CBPARs in more collectivistic communities in low- and middle-income countries, should consider conducting pilot exercises to explore the cultural and traditional practices that could have significant bearing on the IC and research process, and solicit for views from community leaders and members on ways to manage these ethico-cultural challenges in an open and mutually-respectful manner.

### Socioeconomic factors and the IC process in the collectivistic Ghanaian context

Recent developments in medical ethics, together with the growing appreciation of the vast socioeconomic differences across contexts, have heightened the need to revisit the use of the universal ethical IC documentation across groups, particularly among low literacy and highly communal populations [9–12]. Generally, the IC forms and the other research-related information should be written and presented be in a language understandable to prospective participants [5, 41]. In the rural poor African context, where a significant proportion of the population may not be English speakers, it is necessary to translate the consent form into the native language of participants to enhance comprehension. Verbatim English-local dialect translation by research assistants may miss out on important key words and phrases. As may be expected, the majority of individuals in the rural, low-income communities may have had no opportunity for formal education [1]. Prospective participants with low literacy may become anxious and discomfited with the IC process, and the research exercise more generally, stemming from the fear that the demand and expectations from research participants may be beyond their capability, or that they have to sign or complete the IC forms in English, which they do not have the ability to. To address this challenge, the researcher should carefully explain the rationale for the completion of the IC form, including that it serves as evidence of recruitment to the IRBs that approved the study. We do not recommend the use of phrases or explanations that suggest that the IC is a *contract* or *legal* document. A number of participants may be unfamiliar with this construct and may misconstrue that to mean there are legal implications when they consent, or may even associate the IC process with the strategies used by police officers in the colonial era to obtain forced confessions [42]. Of note, the thumb-printing option, which is used in most local, district, regional, and national elections in Ghana and other sub-Saharan African countries, may be more appealing to many individuals in these settings, than signing on the IC form. This should be discussed at an early stage of the IC process as an alternative with prospective participants who may have difficulties with appending signatory to the IC document.

In furtherance, given that some rural poor communities in sub-Saharan Africa are the target and recipients of many social interventions and other supports from state agencies and non-governmental organisations, there is a high possibility that prospective participants (or their relatives) were beneficiaries of asset or cash transfers or other forms of support in previous studies. For instance, in the EP project [39], researchers set out to explore the most cost-effective ways of improving the living standards of very poor individuals, by offering a holistic economic package, which includes an asset transfer, small cash transfers, access to saving, and training and coaching, in addition to the cognitive behavioural and skill development programme for several hundreds of rural poor adult participants. The livelihood empowerment against poverty [43], a flagship programme by the National Social Protection Strategy of Ghana, also provides cash and health insurance to extremely poor households across Ghana with the overarching aim to alleviate short-term poverty and to encourage long term human capital development. We note that previous involvement in interventional studies increases participants’ expectations of receiving some form of reward during or after participating in the study, and can, inadvertently, coerce some individuals to consent to participate in a research study. When obtaining IC from individuals who may have participated in such interventional studies or programmes, there is need to initiate an honest discussion to explain that participants were selected because they fit in the inclusion criteria and that an honest response to the interview questions would be greatly appreciated. This discourse, nonetheless, may not erode the expectations of some individuals, especially those recruited into studies that offer intervention sessions or run for several weeks. Such unresolved expectations may lead prospective participants to make false claims, give socially desirable responses, or provide exaggerated description of their impoverishment in order to qualify for recruitment or support. Nonetheless, in order to build mutual trust and instil a sense of anonymity and confidentiality, we do not recommend statements or explanations that imply that the information they provide would be confirmed with existing dataset, or cross-checked with the chief or elders of the community. A solemn explanation and plea to provide truthful and honest responses should be sufficient.

With large-scale intervention studies that promise some form of incentives, some prospective participants may be more interested in the details of the incentives and may insist on full disclosure before confirming their participation, especially when the period of the research also competes with other important engagements, such as farming—which is the primary source of livelihood for many individuals in these settings. In their quest to inform a prospective participant about the study, the independent mediator, and research assistants in particular, could share project details that may compromise the study, thus becoming a source of undue coercion and deception, albeit unintentionally. This is particularly true in instances where participants would later be randomised into various arms of the study. The temptation to disclose *blinded* information or to *lie* to prospective participants can be alluring to research assistants who need to meet a set target of recruitment, or whose remuneration depends on the number of participants recruited within a given time period. Researchers should discuss with independent mediators and research assistants about the need to refrain from giving false information to prospective participants and encourage them to demonstrate truthfulness and honesty in the IC process. We recommend that supervisors conduct spot-checks for each research assistant—by randomly selecting a few recruited participants to interview them to ascertain the information provided to them about the study during the IC process.

Traditionally, most researchers train their research assistants and surveyors to understand and master the content of the IC documentation, without much consideration of their linguistic abilities of participants’ dialects. A lack of proficiency in participants’ language can pose serious challenges when working with non-English speaking populations. In the rural Ghanaian context, people are more likely to identify and form collaborative relationships with other people who speak the same language or come from the same ethnic or religious group [4]. In addition to professing mastery over the IC material, researchers should be fluent in the native language of the target group in which the IC process, interviews, or intervention programme sessions would be held. Where possible, the research team should engage research assistants who are themselves native speakers of participants’ dialects and are very knowledgeable in the cultural customs of the people. It is also possible for prospective participants from remote, highly collectivistic settings to view researchers who have strong, unfamiliar accent as strangers or foreigners, and become sceptical of the research activity. Whether or not research assistants are native speakers, it is essential that the entire IC form, or key phrases on the form, are translated into participants’ native language before they are administered.

### The Declaration of Helsinki and its implications for social science research

One of the original motivating factors in developing UEGs for medico-social research was the significant number of unethical researches conducted in the early 1960s (e.g., the Thalidomide Drug Tragedy, the Tuskegee Syphilis Study, the Willowbrook Hepatitis Study, Zimbardo’s Prison Study) [44]. These events led to the development of guidelines to protect human lives and dignity and to prevent the exploitation of study participants, especially minority groups. Typically, UEGs provide a common set of principles/values carefully selected to guide researchers and practitioners in their engagement with study participants or clients. Adopted by the World Medical Association in 1964 at its 18^th^ general meeting in Helsinki, Finland, the Declaration of Helsinki (DoH) [2, 8] is by far the most widely used statements of ethical principles that provide ethical standards and clinical governance for the conduct of *medical* research [45]. The current version (2013), which is its seventh revision, has an expanded session on the IC process that partly reads: “…*Although it may be appropriate to consult family members or community leaders, no individual capable of giving informed consent may be enrolled in a research study unless he or she freely agrees*.” (p. 3). This addition has important implications for the IC process. First, we note, by this statement that the DoH does not deny the possibility that a researcher (or prospective participant) may need to involve family or community members in the IC process at a point. Of note, this guideline differs from the guideline requiring that the IC be completed by a legally acceptable representative, occasioned by the incapacity or vulnerability of the prospective participant. By the concluding phrase, the DoH however insists that a prospective participant who is not under age or does not have any form of incapacity should be able to *finalise* their decision of whether to participate or not—by themselves. We note that the DoH is silent on the *process* of this engagement with family or community members, probably for reasons of space.

Second, we note that the events leading to the establishment of the DoH were largely due to the conduct of controversial and harmful biomedical studies that came to public attention. The primordial, overarching aim of the DoH *is* to develop a framework that supports a more ethical approach of conducting medical research in a way and manner that prevent harm, maximise the benefits from the findings, and preserves human dignity. Although a significant number of social science researchers and research institutions base aspects of their research ethics on the DoH, there are three reasons to believe that the DoH does not provide *sufficient* guideline for the conduct of social science research. First, a significant number of medical research does involve some level of risk or discomfort that may involve an invasive procedure. Often the implications of these procedures are undetermined from the beginning. Consider the initial human clinical trial (Phase 1) of the recently developed Covid-19 vaccines. Here, tens and hundreds of healthy individuals were recruited, vaccinated, and monitored to assess the initial safety of the vaccine and to identify the correct dose. At this level of the trial, scientists *do* expect some adverse effects of the vaccine, but are unable to predict the exact effects. This sort of study, indeed, requires that participants are fully informed about the possible implications and be allowed to take informed decision about whether or not to participate. While the decisional authority of the individual is paramount in this regard, others may also argue that married individuals who decide to participate in such a trial *must* obtain approval from their spouses—since scientists could not tell if any of the possible adverse effects could affect their health, including their sexual function. Second, the DoH and related UEGs are premised on the individualistic autonomy, where the decisional authority rests solely on the individual. However, among some ethnic groups in Ghana, as discussed previously, it may be inappropriate for a married female adult in sound mind and without any incapacity to take a sole decision on such more or less important issue as participating in a research—without the consent of her husband. This complexity, no doubt, contradicts the principle of autonomy, yet, cannot be circumvented in these contexts.

Third, typical with all UEGs, the DoH does not claim to provide a complete, comprehensive guidelines capable of resolving ethico-cultural conflicts for researchers in all possible situations that will arise. This limitation, by itself, suggests that the DoH and all other UEGs, for that matter, cannot provide a universal, one-size-fits-all document, particularly for researchers working in more collectivistic, low literate African settings. Because the DoH holds such importance in some aspects of social science research (e.g., cognitive and neuropsychology research), we ignore the issue of whether it is necessary or should be the ultimate guide for social science researchers who apply the CBPAR approach in their research settings. It is our considered view that the DoH, which is relied on and quoted in support of ethical research in research protocols of social sciences in some Universities and research institutions, is not a sufficient guide for general social science research. We observe that some countries and Universities have adapted the principles of the DoH and tailored them to their specific contexts. We posit that what is needed is a broader conceptualisation of an IC process that allows researchers to manage ethico-cultural conflicts with the IC more appropriately in their research settings, in circumstances when UEGs are silent or take a contrary stance. We contend that within a multi-stakeholder approach comprising local communities, Universities, research institutions, IRBs, and research-based NGOs, it should be possible to build on existing knowledge [31–34], and on recent community-based research efforts in Ghana [1, 40, 46–48] to develop a framework that reflects and takes into account the general perspective and cultural norms of the Ghanaian peoples, as well as the principles of the UEGs.

Although we do not question the principle of individual autonomy or the need for vigorous IC policy, we argue that the IC process for research participants should provide participants with adequate information about the study in a language and at the level of their understanding, and should also contribute to minimising the incidence of deception and coercion. Our view is informed by research and field observations [3, 5, 25, 40, 46–48], and by Kant’s [49] conceptualisation of autonomy as a medium by which individuals freely exercise their practical reasoning, which also provides them the forum to safeguard and protect themselves against coercion and deception. The IC process and its documentation, as well as guidelines for the conduct of social science research in the more communal sub-Saharan African context, should go beyond ensuring the autonomy and decisional authority of prospective participants, to also include mechanisms to prevent deception and coercion.

## Conclusions

It is becoming increasingly difficult to ignore the ethico-cultural and methodological challenges associated with applying the UEGs, which is principled on the individualistic autonomy, in the rural, more collectivistic, low literate communities in sub-Saharan Africa. While these guidelines are appropriate for conducting research with individuals from Western, educated, industrialised, rich, and democratic societies, there are some complexities when using this framework for CBPAR in highly communal African contexts. For instance, how can researchers accommodate the cultural roles of gatekeepers such as the chief and his elders, heads of households, and the husbands of female prospective participants during information disclosure and the entire IC process? Should the process for obtaining the IC rest entirely on the independent mediators, and to what degree do they influence voluntariness? In what ways can researchers prevent or minimise deception and coercion of prospective participants in these settings?

In order to proactively empower emancipatory research in these settings, it should be possible—and in fact, mandatory—for researchers to adopt an approach that preserves the scientific rigour and insights from the universal ethical protocols, that also accommodates and adheres to the cultural customs and practices in the local context. We urge IRBs to encourage researchers proposing CBPARs in highly communitarian, low literate African communities to explore and discuss in their protocols some ethico-cultural delimmas and conflicts they envision in their target population, and how they intend to manage them, while also safeguarding the scientific principles underpinning the research process. For instance, in settings where participants are unable to sign on the IC forms (due to low literacy levels), or where cultural practices emphasise on oral rather than written agreements, we proffer that it should be sufficient for the researcher to obtain an oral approval, which should be documented and witnessed by the independent mediator or any other member of the household who is able and willing to.

We hasten to state that our views and suggestions should not, by any means, be interpreted to mean that we embolden researchers in the described settings to waive or disregard the traditional (universal) research IC guidelines. This piece should be considered, instead, as an addendum to the ongoing discussions and suggestions [5, 9–12] to re-examine the use of the UEGs in order to prevent ethics dumping and safeguard the rights of research participants in local communities in sub-Saharan Africa. In the long term, there may be a need, in our view, to develop an African-centred research ethical guidelines for social scientists and researchers working in the described contexts. Such a protocol should necessarily be built on, and embrace the theoretical principles underpinning the traditional scientific research methods, as well as on the traditional practices and beliefs of the local communities that wield significant influence on their social lives. Who else can lead this course other than our Universities, research institutions, and IRBs? Although we do not intend for this discourse to exhaust this important subject in a complete way, it is our hope that these discussions will stimulate others to reflect upon the challenges with applying the IC in the more communal, rural context of Ghana, and sub-Saharan Africa more generally—and propose suitable, diversified, pluriversal, context-specific alternatives.

## Abbreviations

CBPAR: Community-based participatory action research
IC: Informed consent
ILP: Inspired life programme
IPA: Innovations for poverty action
IRB: Institutional Review Board

## Footnotes

1 A person tasked or employed by community leaders to make public announcements in the streets or marketplace of a town.
